# Biopharmaceutical Characterization of Grapiprant Within the BCS Framework Under Canine-Relevant Conditions: Solubility and Caco-2 Permeability Assessment

**DOI:** 10.3390/pharmaceutics18081010

**Published:** 2026-08-15

**Authors:** Zeyu Wen, Xilu Sun, Sumeng Chen, Jinyan Meng, Runlin Yu, Shuyan Guo, Xingyuan Cao

**Affiliations:** 1 College of Veterinary Medicine, China Agricultural University, Beijing 100193, China; wenzy1124@163.com (Z.W.); 18796119112@163.com (X.S.); phdchensumeng@163.com (S.C.); jinyanmeng00@outlook.com (J.M.); yurunliny@163.com (R.Y.); guoshuyan1566@163.com (S.G.); 2 National Center for Veterinary Drug Safety Evaluation GCP Laboratory, Beijing 100193, China

**Keywords:** grapiprant, solubility, permeability, biopharmaceutical properties, Caco-2 cell monolayer model

## Abstract

**Background**: The Biopharmaceutics Classification System (BCS) classifies drug substances according to their aqueous solubility and intestinal permeability; however, its application to veterinary drugs should account for species-specific gastrointestinal physiology. Grapiprant is a selective prostaglandin E_2_ receptor subtype 4 (EP4) antagonist approved as an oral tablet to control pain and inflammation associated with osteoarthritis in dogs, but its properties within the BCS framework remain unclear. **Methods**: This study evaluated the equilibrium solubility of grapiprant across pH conditions relevant to the canine gastrointestinal tract and calculated the dose number (D_0_) based on different gastric fluid volumes. A Caco-2 cell monolayer model was used to assess grapiprant intestinal permeability and the effects of time, concentration, pH, and efflux transporter inhibitors on its transepithelial transport. **Results**: Grapiprant showed relatively small changes in solubility across the tested pH range. D_0_ varied with pH and gastric fluid volume and approached or fell below 1 at larger fluid volumes. In the Caco-2 model, grapiprant showed generally limited apparent absorptive permeability, with permeability varying with pH. Efflux ratios were greater than 1, and verapamil reduced the efflux ratio, suggesting possible P-glycoprotein (P-gp) involvement. **Conclusions**: These findings suggest that both solubility and apparent intestinal permeability may limit the oral absorption of grapiprant under certain canine-relevant conditions. Accordingly, this study provides a biopharmaceutical characterization of grapiprant within the BCS framework rather than a definitive canine BCS classification, highlighting the importance of considering canine gastrointestinal conditions when interpreting its solubility and permeability. The results may support future optimization of dosing conditions and oral formulations.

## 1. Introduction

**Figure 1:**
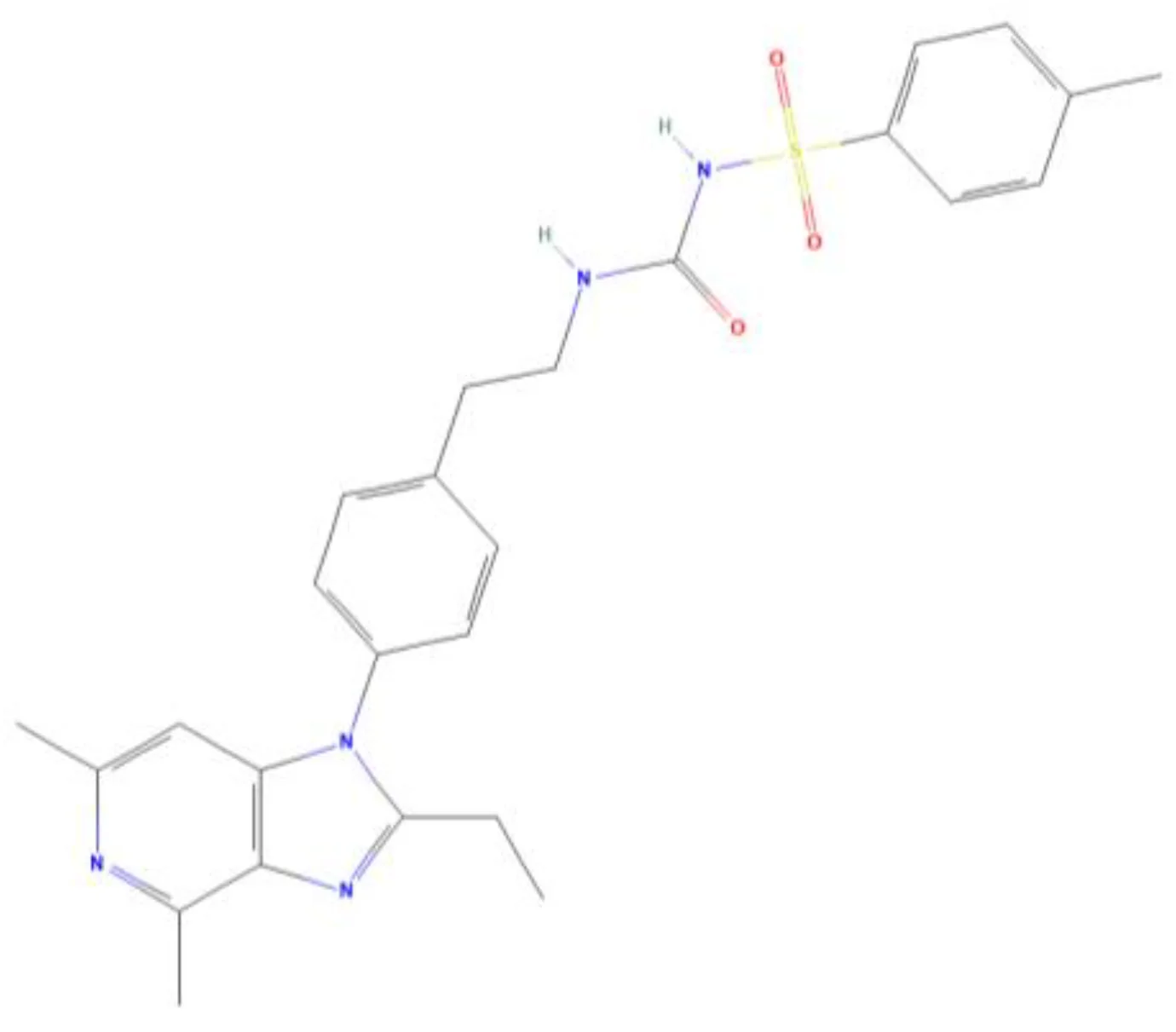
Two-dimensional chemical structure of grapiprant obtained from PubChem (CID 11677589).

Grapiprant is a selective antagonist of prostaglandin E_2_ (PGE_2_) receptor subtype 4 (EP4) and exerts analgesic effects by blocking PGE_2_-EP4 signaling involved in inflammation and pain (the chemical structure is shown in Figure 1) [1,2]. Unlike conventional nonsteroidal anti-inflammatory drugs (NSAIDs), which non-selectively inhibit cyclooxygenase (COX) enzymes, grapiprant reduces interference with normal COX-mediated physiological functions, thereby potentially providing an improved safety profile [3,4]. Based on these characteristics, grapiprant has been approved for the management of osteoarthritis-associated pain in dogs, providing a new therapeutic option for chronic pain control in dogs [5,6,7].

Oral administration is the most common route of drug delivery in dogs due to its convenience, ease of administration, and good clinical applicability. Grapiprant is currently marketed as an oral tablet formulation for canine use, available in three strengths of 20, 60, and 100 mg per tablet. However, its therapeutic efficacy is not only associated with its EP4 receptor antagonistic activity but also depends on sufficient and consistent systemic exposure after oral administration. The latter is primarily influenced by the drug’s physicochemical properties, formulation characteristics, and physiological factors of the canine gastrointestinal tract, including gastrointestinal (GI) transit time, absorptive surface area, active transporters, and metabolizing enzymes in enterocytes and the liver [8]. These factors may affect oral absorption by influencing drug dissolution, intestinal transport, and metabolism. Therefore, characterization of the biopharmaceutical properties of grapiprant, particularly its solubility and intestinal permeability, is essential for understanding its oral absorption behavior and variations in systemic exposure [9,10,11].

The Biopharmaceutics Classification System (BCS) categorizes drug substances into four classes based on aqueous solubility and intestinal permeability, two key parameters that influence the rate and extent of oral drug absorption and provide insights into drug bioavailability [12,13,14]. Originally developed for human pharmaceutical compounds, the BCS provides an important framework for evaluating oral drug absorption and guiding formulation development. However, the criteria established under human conditions may not be directly applicable to veterinary species [13]. Nevertheless, the BCS framework remains useful for veterinary biopharmaceutical evaluation when species-specific gastrointestinal physiology and relevant assessment conditions are considered. Within the BCS framework, drug solubility is commonly evaluated using the dose number (D_0_), which considers the dose strength, gastrointestinal fluid volume, and drug solubility. The fluid volume used for D_0_ calculation is generally based on a human reference volume of 250 mL. However, for veterinary species such as dogs and cats, the volume of gastrointestinal fluid under fasting conditions is influenced not only by residual gastric fluid but also by factors including water intake, body size, breed, and physiological differences in the gastrointestinal tract [8,15]. Therefore, solubility assessments based on human gastrointestinal conditions should not be directly extrapolated to veterinary species without considering species-specific physiological conditions. In addition, differences between fed and fasted states can influence gastrointestinal pH, bile salt availability, and luminal fluid composition, thereby affecting drug solubility and dissolution behavior within the gastrointestinal tract [16,17,18]. Therefore, considering canine-specific physiological conditions, characterization of the solubility profile of grapiprant under relevant gastrointestinal conditions is essential for understanding its oral absorption process and variations in systemic exposure.

Adequate drug solubility in gastrointestinal fluids is a prerequisite for oral absorption, and its ability to further cross the intestinal epithelial barrier and enter the systemic circulation depends on intestinal permeability [19,20,21]. Currently, approaches for evaluating intestinal permeability mainly include in vitro cell models, ex vivo intestinal tissue permeability assays, parallel artificial membrane permeability assays, and in vivo animal studies [22,23,24]. Among these approaches, in vitro cell models are widely used because they allow transepithelial transport and potential transport mechanisms to be evaluated under controlled and reproducible conditions. More physiologically representative approaches, such as in situ intestinal perfusion and ex vivo intestinal tissue models, can better reflect native intestinal conditions but involve greater technical and practical constraints. Specifically, in situ perfusion requires anesthesia, surgery, and intestinal cannulation, whereas ex vivo assays rely on freshly excised tissue with preserved epithelial viability and barrier integrity and are sensitive to tissue source and handling [25,26]. Canine intestinal organoid-derived monolayers offer greater species relevance, but their use in drug permeability assessment remains at the proof-of-concept stage. Establishing these models requires primary canine intestinal tissue, expansion and differentiation of three-dimensional organoids, and further optimization to generate reproducible monolayers on permeable supports.

Given the practical and methodological limitations of the more physiologically or species-relevant approaches described above, human colorectal adenocarcinoma cells (Caco-2) and Madin–Darby canine kidney (MDCK) cells are frequently used monolayer models for in vitro permeability evaluation [27]. Among these models, Caco-2 cells can spontaneously differentiate into polarized monolayers with intestinal epithelial-like characteristics under in vitro culture conditions. Their morphological features, barrier functions, and permeability properties exhibit certain similarities to those of small intestinal columnar epithelial cells, making them widely used for evaluating intestinal permeability and transmembrane transport characteristics of drugs [28,29]. Accordingly, the Caco-2 model was selected in the present study because it is well established and allows transport experiments to be conducted under controlled and reproducible conditions. Determination of the apparent permeability coefficient (Papp) of grapiprant across Caco-2 monolayers, combined with bidirectional transport studies and calculation of the efflux ratio (ER), was used to characterize its apparent transepithelial permeability and potential efflux characteristics. Together, these measurements provided experimental evidence for the biopharmaceutical characterization of grapiprant within the BCS framework.

Several studies have investigated the pharmacokinetic characteristics of grapiprant in dogs, including systemic exposure and bioavailability following single and multiple oral administrations [30,31,32]. However, existing findings suggest that the oral exposure of grapiprant may be influenced by multiple factors. The absolute bioavailability of grapiprant in dogs was reported to be approximately 62% at a dose of 1 mg/kg [33]. In addition, differences in bioavailability have been observed between fasted and fed dogs following oral administration of grapiprant [34]. These findings indicate that the oral exposure of grapiprant may vary under different dosing conditions, although the factors contributing to these differences remain unclear.

Although these pharmacokinetic studies have provided important information for understanding the absorption and exposure characteristics of grapiprant, information on its biopharmaceutical properties remains limited, particularly regarding its solubility and intestinal permeability under canine-relevant conditions within the BCS framework. Therefore, the present study aimed to characterize the biopharmaceutical properties of grapiprant by evaluating its solubility under canine-relevant gastrointestinal conditions and its intestinal transport using a Caco-2 cell monolayer model. The findings of this study provide experimental evidence for the biopharmaceutical characterization of grapiprant within the BCS framework and may contribute to a better understanding of the potential factors influencing its oral absorption in dogs. Furthermore, these findings may inform future optimization of grapiprant oral formulations.

## 2. Materials and Methods

### 2.1. Chemicals and Reagents

Caco-2 cells, phosphate-buffered saline (PBS), Caco-2 complete culture medium, fetal bovine serum, trypsin, and Hank’s Balanced Salt Solution (HBSS) were purchased from Shanghai Yizefeng Biotechnology Co., Ltd. (Shanghai, China). Grapiprant active pharmaceutical ingredient (purity: 98%) was provided by Luoyang Huizhong Veterinary Drug Co., Ltd. (Luoyang, Henan, China). The analytical reference standard of grapiprant (purity: 99.3%) was purchased from Shanghai Yuanye Bio-Technology Co., Ltd. (Shanghai, China). The internal standard, trazodone (purity: 98%), was obtained from Aladdin Industrial Corporation (Shanghai, China). Dimethyl sulfoxide (cell culture grade; DMSO), sodium fluorescein, the P-glycoprotein (P-gp) inhibitor verapamil hydrochloride, the multidrug resistance-associated protein 2 (MRP2) inhibitor probenecid, and the breast cancer resistance protein (BCRP) inhibitor reserpine were purchased from Beijing Solarbio Science & Technology Co., Ltd. (Beijing, China). Cell lysis buffer, enhanced Cell Counting Kit-8 (CCK-8), an alkaline phosphatase (ALP) assay kit, and BCA protein concentration assay kit were purchased from Beyotime Biotechnology Co., Ltd. (Shanghai, China). HPLC-grade methanol (MeOH) and formic acid (FA) were obtained from Thermo Fisher Scientific (Macquarie Park, NSW, Australia). Ultrapure water was prepared using a Milli-Q purification system (Merck Millipore, Burlington, MA, USA). Transwell plates (12 well format, 0.4 μm pore size, polycarbonate membrane) were purchased from Corning Incorporated (Corning, NY, USA). Chromatographic separation was performed using a ZORBAX SB-Aq C18 column (4.6 mm × 250 mm, 5 μm; Agilent Technologies, Santa Clara, CA, USA) and an ACQUITY UPLC BEH C18 column (2.1 mm × 50 mm, 1.7 μm; Waters Corporation, Milford, MA, USA).

### 2.2. Determination of Equilibrium Solubility

Saturated solutions of grapiprant in buffer media with different pH values ranging from 1.6 to 8.0 were prepared in 50 mL centrifuge tubes by adding an excess amount of the drug until saturation was achieved, which was indicated by the presence of visible undissolved drug particles. The mixture was shaken for 24 h in a thermostatic water bath shaker at 38.5 ± 0.5 °C and 150 rpm, with the temperature selected to approximate the physiological body temperature of dogs. The mixture was then allowed to stand for 2 h. The supernatant was collected, filtered through a 0.22 μm membrane filter, and diluted with the corresponding pH buffer. The equilibrium solubility of grapiprant was determined by HPLC. For each pH condition, three replicate samples were prepared and analyzed.

D_0_ was calculated from the equilibrium solubility data using Equation (1) to evaluate the solubility classification of grapiprant.

(1) $$D_{0}=\frac{M/V_{0}}{C_{S}}$$

where M is the maximum administered dose (mg), V_0_ is the gastric fluid volume in dogs (mL), and Cs is the equilibrium solubility of the drug (mg/mL). A drug was classified as highly soluble when D_0_ ≤ 1 and as having low solubility when D_0_ > 1 [8].

### 2.3. Cell Culture and Cytotoxicity Assay

Caco-2 cells were maintained in complete medium at 37 °C in a 5% CO_2_ incubator. Upon reaching 80% confluence, the medium was removed, and the cells were gently rinsed with sterile PBS. Trypsin was added for cell detachment at 37 °C for 3 min, and the reaction was terminated by adding complete medium. The cells were then centrifuged at 1000 rpm for 5 min, resuspended in fresh medium, and passaged at a 1:3 ratio.

The cytotoxicity of grapiprant was evaluated using a CCK-8 assay. Cells were seeded into 96-well plates at a density of 2 × 10^5^ cells/mL and allowed to adhere. Grapiprant was dissolved in DMSO and diluted with complete medium to final concentrations ranging from 1.56 to 50 μg/mL, with a final DMSO concentration of 0.1%. Following treatment for 6, 12, 24, or 48 h, CCK-8 reagent was then added to each well, followed by incubation for 1 h at 37 °C. Absorbance was measured at 450 nm. Cell viability was calculated according to Equation (2).

(2) $$Cell\;viability\;\left(\%\right)=\frac{{OD}_{test}-{OD}_{blank}}{{OD}_{control}-{OD}_{blank}}\times 100$$

where OD_test_, OD_blank_, and OD_control_ represent the absorbance values of grapiprant-treated wells, blank wells containing no cells, and vehicle-control wells containing cells treated with 0.1% DMSO, respectively.

### 2.4. Establishment of the Caco-2 Cell Monolayer Model

Caco-2 cells were seeded onto Transwell inserts in 12-well plates by adding 0.5 mL of cell suspension at a density of 2 × 10^5^ cells/mL to the apical (AP) side, corresponding to 1 × 10^5^ cells per insert. A total of 1.5 mL of complete medium was added to the basolateral (BL) side. Cells were cultured at 37 °C. The medium was renewed every other day for the first 7 days and daily thereafter. Cell monolayer formation was monitored by light microscopic observation.

### 2.5. Evaluation of the Caco-2 Cell Monolayers

#### 2.5.1. Morphological Observation of Caco-2 Cells

Cell morphology was monitored regularly by light microscopy during the 21-day culture period. For ultrastructural analysis, selected monolayers were rinsed with PBS, fixed with 2.5% glutaraldehyde at 4 °C overnight, and subsequently processed for transmission electron microscopy (TEM).

#### 2.5.2. Measurement of Transepithelial Electrical Resistance

Transepithelial electrical resistance (TEER) was measured on days 1, 7, 14, and 21 using a cell resistance meter to assess monolayer integrity. The electrodes were disinfected with 70% ethanol for 15 min, air-dried, and equilibrated in prewarmed HBSS at 37 °C for 15 min. Prior to measurement, the culture medium was replaced with 0.5 mL and 1.5 mL of prewarmed HBSS in the AP and BL sides, respectively, followed by equilibration in the incubator for 15 min. Three readings were taken at different positions in each well, and TEER was calculated according to Equation (3).

(3) $$TEER=\left(R_{test}-R_{blank}\right)\times A$$

where R_test_ is the resistance of the insert containing the cell monolayer (Ω), R_blank_ is the resistance of a cell-free insert containing the same medium (Ω), and A is the effective membrane area of the Transwell insert (1.12 cm^2^). Monolayers with TEER values greater than 500 Ω·cm^2^ were considered intact and used for subsequent experiments.

#### 2.5.3. Determination of Alkaline Phosphatase Activity

ALP activity was measured on days 7, 14, and 21 to evaluate cell polarization. The culture medium was aspirated, and the monolayers were rinsed three times with HBSS. Prewarmed HBSS was then added to the AP and BL sides at volumes of 0.5 and 1.5 mL, respectively, followed by incubation for 30 min. HBSS samples were collected from both sides, and ALP activity was determined using an ALP assay kit.

#### 2.5.4. Sodium Fluorescein Permeability Assay

Monolayers with TEER ≥ 500 Ω·cm^2^ were selected for the permeability assay. After rinsing with prewarmed HBSS and equilibration for 30 min, 0.5 mL of HBSS containing 200 μg/mL sodium fluorescein was added to the AP side, and 1.5 mL of blank HBSS was added to the BL side. The plates were incubated for 90 min, after which samples were collected from the BL side. Standard solutions of sodium fluorescein at concentrations of 0.125, 0.25, 0.5, 1, 2, and 4 μg/mL were prepared, and absorbance was measured at 490 nm to generate a standard curve.

### 2.6. Caco-2 Cell Transport Experiment

#### 2.6.1. Effects of Time and Concentration on Grapiprant Transport

Caco-2 cell monolayers that had been successfully established were used for the bidirectional transport assay of grapiprant. Before the experiment, the cell monolayers were gently rinsed three times with HBSS prewarmed to 37 °C. Prewarmed HBSS was then added to the AP and BL sides at volumes of 0.5 mL and 1.5 mL, respectively, and the monolayers were equilibrated in an incubator for 30 min. After equilibration, the HBSS was removed from both sides.

Grapiprant transport solutions were prepared by diluting a 50 μg/mL stock solution with HBSS to final concentrations of 1, 2.5, 5, 10, 25, and 50 μg/mL. All transport solutions were sterilized by filtration through a 0.22 μm membrane before use.

For transport from the AP side to the BL side, 0.5 mL of HBSS containing grapiprant at different concentrations was added to the AP side, while 1.5 mL of blank HBSS was added to the BL side. Samples of 300 μL were collected from the BL side at 15, 30, 45, 60, and 90 min, and an equal volume of blank HBSS prewarmed to 37 °C was immediately replenished. For transport from the BL side to the AP side, 1.5 mL of HBSS containing grapiprant at different concentrations was added to the BL side, while 0.5 mL of blank HBSS was added to the AP side. Samples were collected at the same time points, with 100 μL taken from the AP side each time, followed by immediate replacement with an equal volume of blank HBSS prewarmed to 37 °C.

During the experiment, the culture plates were continuously incubated at 37 °C. The collected samples were stored at -20 °C until analysis. After sample preparation as described in Section 2.9, grapiprant concentrations were determined by UPLC-MS/MS. The cumulative transport amount (Q), Papp, and ER were calculated using the following Equations (4)–(7).

Considering the dilution of the drug concentration on the receiver side after sampling at each time point, the cumulative amount of drug transported was calculated as follows:

(4) $$Q_{\left(AP-BL\right)}=1.5C_{n}+0.3\sum_{i}C_{i}\left(i=n-1\right)$$

(5) $$Q_{\left(BL-AP\right)}=0.5C_{n}+0.1\sum_{i}C_{i}\left(i=n-1\right)$$

where Q_(AP-BL)_ is the cumulative amount transported from the AP side to the BL side (ng/mL); Q_(BL-AP)_ is the cumulative amount transported from the BL side to the AP side (ng/mL); and Cn is the transported concentration in the sample.

(6) $$Papp=\left(dQ/dt\right)/\left(A\times C_{0}\right)$$

where dQ/dt is the drug transport rate per unit time (ng/s), determined as the slope of the linear portion of the cumulative transport amount-time curve by linear regression over the 15–90 min interval; A is the effective membrane area of the Transwell insert, with A = 1.12 cm^2^; and C0 is the initial drug concentration on the donor side (ng/mL). The linearity of the cumulative transported amount–time relationship was evaluated using the coefficient of determination (R^2^).

(7) $$ER={Papp\;}_{\left(BL-AP\right)}/{Papp\;}_{\left(AP-BL\right)}$$

where Papp _(BL-AP)_ is the permeability coefficient from the BL side to the AP side (cm/s), and Papp _(AP-BL)_ is the permeability coefficient from the AP side to the BL side (cm/s).

#### 2.6.2. Effects of pH on Grapiprant Transport

To evaluate the effect of pH on grapiprant transport, HBSS was adjusted to pH 6.3, 7.2, or 8.0 using 0.1 mM HCl or 0.1 mM NaOH, and then sterilized by filtration through a 0.22 μm membrane. Grapiprant solutions at 10 μg/mL were prepared by diluting the 50 μg/mL grapiprant solution with HBSS at the corresponding pH. After each sampling, an equal volume of HBSS with the corresponding pH, prewarmed to 37 °C, was immediately added. The remaining procedures were performed as described in Section 2.6.1, and Papp and ER were calculated.

#### 2.6.3. Effects of Efflux Inhibitors on Grapiprant Transport

To evaluate the effects of efflux inhibitors on grapiprant transport, verapamil hydrochloride, probenecid, and reserpine were prepared in HBSS containing 0.1% DMSO at concentrations of 100 μM, 2 mM, and 100 μM, respectively, and sterilized by filtration through a 0.22 μm membrane. Before the transport assay, the corresponding inhibitor solution was added to both the AP and BL sides of the Caco-2 cell monolayers, followed by equilibration at 37 °C for 30 min.

For the AP-BL transport direction, 0.5 mL of HBSS containing grapiprant (10 μg/mL) and the corresponding inhibitor was added to the AP side, while 1.5 mL of HBSS containing the corresponding inhibitor was added to the BL side. For the BL-AP transport direction, 1.5 mL of HBSS containing grapiprant (10 μg/mL) and the corresponding inhibitor was added to the BL side, while 0.5 mL of HBSS containing the corresponding inhibitor was added to the AP side. After each sampling, an equal volume of HBSS containing the corresponding inhibitor and prewarmed to 37 °C was immediately replenished. The remaining procedures were performed as described in Section 2.6.1, and Papp and ER were calculated.

### 2.7. Caco-2 Cell Uptake Experiment

When evaluating the effects of concentration and pH on grapiprant uptake in the Caco-2 cell monolayer model, the uptake assay was performed simultaneously with the transport assay. After sample collection in the transport assay, the cell culture plates were immediately placed on ice. The cells were gently washed three times with prechilled PBS, followed by the addition of 200 μL of cell lysis buffer to each well. After standing for 15 min, the cells were gently scraped with a cell scraper and transferred to 1.5 mL centrifuge tubes. The cell suspension was disrupted by ultrasonication for 10 cycles and centrifuged at 12,000 rpm for 5 min at 4 °C. The supernatant was collected and stored at −20 °C until analysis. An aliquot of the supernatant was processed as described in Section 2.9, and the grapiprant concentration was determined by UPLC-MS/MS. Another aliquot was used to determine the cellular protein content using a BCA protein concentration assay kit. The cellular uptake amount (U) was calculated using the following Equation (8).

(8) $$U=C/C_{protein}$$

where C is the concentration of grapiprant in the cell sample; C_protein_ is the cellular protein concentration; and U denotes the uptake amount, with a unit of mg/g, corresponding to the mass of grapiprant per gram of protein.

### 2.8. Distribution and Mass Balance of Grapiprant in the Caco-2 Transport System

The distribution of grapiprant in the Caco-2 transport system was evaluated at the end of the experiment. The amounts of grapiprant remaining in the donor side (Q_D,final_), detected in the receiver side (Q_R,final_), and measured as cellular uptake in Caco-2 cells (Q_cell,final_) were quantified and expressed as percentages of the initial amount added to the donor side at 0 h (QD,0H ). The mass balance (%) was calculated using Equation (9).

(9) $$Mass\;balance\left(\%\right)=\frac{\left(Q_{D,final}+Q_{R,final}+Q_{cell,final}\right)}{Q_{D,0H}}\times 100\%$$

Under all experimental conditions, the mass balance of grapiprant was greater than 80%, indicating acceptable recovery of grapiprant in the Caco-2 transport system and supporting the reliability of the transport and uptake data.

### 2.9. Sample Preparation

A 90 μL aliquot of the cell transport solution was transferred to a 1.5 mL centrifuge tube, followed by the addition of 10 μL of the internal standard working solution (1 μg/mL) prepared in 50% methanol. The mixture was vortexed for 5 min and centrifuged at 12,000 rpm for 10 min at 4 °C. An 80 μL aliquot of the supernatant was then transferred to an autosampler vial for UPLC-MS/MS analysis.

### 2.10. HPLC Conditions

Chromatographic separation of samples from the equilibrium solubility assay was performed on a ZORBAX (Agilent Technologies, Santa Clara, CA, USA) SB-Aq C18 column (4.6 mm × 250 mm, 5 μm) at a flow rate of 1 mL/min, with the column temperature maintained at 40 °C. The mobile phases consisted of water (A) and methanol (B), and the separation was performed under isocratic elution at an A:B ratio of 1:1.

### 2.11. UPLC-MS/MS Conditions

Multiple-reaction monitoring (MRM) in positive-ion electrospray ionization (ESI) mode was performed on the UPLC-MS/MS system for drug quantification. The cell samples were injected and separated on an ACQUITY UPLC BEH C18 column (2.1 mm × 50 mm, 1.7 μm) at a flow rate of 0.3 mL/min and a column temperature of 35 °C. The mobile phases consisted of (A) 0.1% formic acid in water and (B) 0.1% formic acid in methanol, delivered according to the following gradient program: 10% B from 0 to 1.5 min; 10–80% B from 1.5 to 2.0 min; 80% B from 2.0 to 4.0 min; 80–10% B from 4.0 to 5.0 min; 10% B from 5.0 to 5.5 min.

### 2.12. Method Validation

The method was validated for specificity, linearity, accuracy, precision, and other relevant parameters in accordance with the Technical Guideline for the Validation of Quantitative Analysis Methods of Biological Samples.

### 2.13. Data Analysis

Statistical analyses were performed using GraphPad Prism (version 10.4.0, GraphPad Software, Boston, MA, USA). Data are presented as the mean ± standard deviation (SD). At each time point, paired measurements from the AP and BL sides were compared using the Wilcoxon matched-pairs signed-rank test. Comparisons among multiple groups were performed using one-way analysis of variance (ANOVA), followed by Dunnett’s multiple-comparisons test when groups were compared with a designated control group, or Tukey’s multiple-comparisons test when all pairwise comparisons were performed. A *p*-value < 0.05 was considered statistically significant.

## 3. Results

### 3.1. Results of Method Validation

Analytical methods were established and validated for the determination of grapiprant in samples from the equilibrium solubility study and Caco-2 cell transport study using HPLC and UPLC-MS/MS, respectively. The validation parameters included linearity, intra- and inter-batch precision, and accuracy.

Both methods showed good linearity over three consecutive days (R^2^ > 0.99), within concentration ranges of 10–200 μg/mL for HPLC and 1–1000 ng/mL for UPLC-MS/MS. For HPLC, intra- and inter-batch precision values were ≤3.72% at the LQC, MQC, and HQC levels and ≤4.07% at the LLOQ, with accuracy bias ranging from 6.22% to 10.99% and from −13.89% to 3.55%, respectively. The corresponding precision values for UPLC-MS/MS were ≤7.78% and ≤7.89%, with accuracy bias ranging from −14.32% to 13.27% and from −8.52% to 14.56%, respectively. All results met the acceptance criteria for the analytical method.

### 3.2. Effect of pH on Grapiprant Solubility

The solubility of grapiprant exhibited a pH-dependent profile across the investigated pH range (1.6–8.0) (Table 1). The solubility gradually decreased from 732.07 ± 5.53 μg/mL at pH 1.6 to the lowest value of 546.84 ± 19.22 μg/mL at pH 5.87, followed by a gradual increase under more alkaline conditions, reaching 686.94 ± 1.21 μg/mL at pH 8.0.

Considering the influence of canine gastrointestinal fluid volume on BCS-based solubility assessment, the dose number (D_0_) values of grapiprant were calculated using different fluid volume conditions (6, 24, and 35 mL) and pH conditions (Table 2). The calculated D_0_ values ranged from 0.78 to 6.10. Under the 6 mL and 24 mL fluid volume conditions, the D_0_ values remained above 1 across all tested pH conditions. In contrast, when the fluid volume was increased to 35 mL, the D_0_ values ranged from 0.78 to 1.05, with several pH conditions showing D_0_ values close to or below 1.

### 3.3. Effect of Grapiprant on Caco-2 Cell Viability

**Figure 2:**
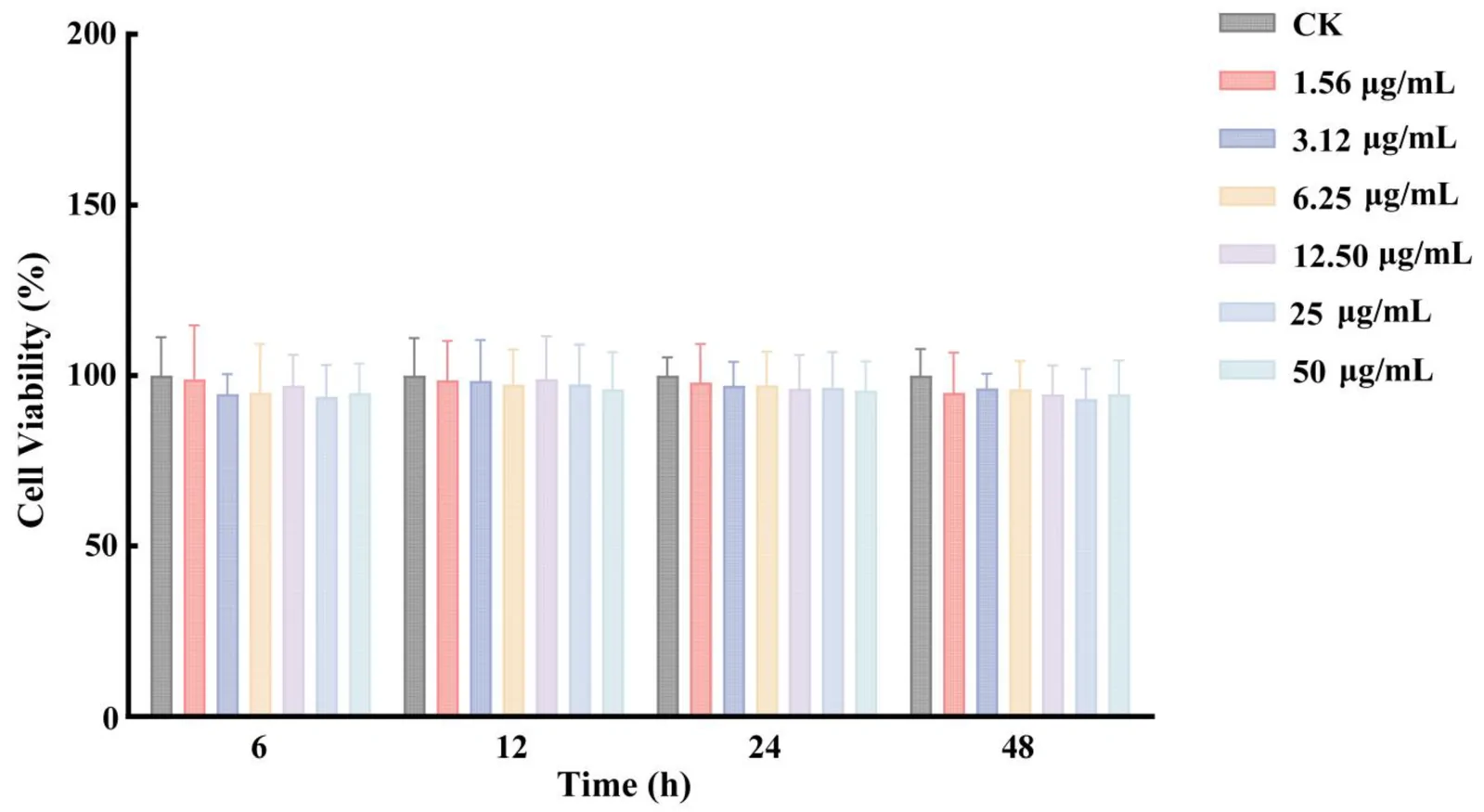
Effects of grapiprant on Caco-2 cell viability measured by the CCK-8 assay. Significance test: At each time point, one-way ANOVA followed by Dunnett’s multiple-comparisons test was conducted, with the control group serving as the reference.

The cytotoxicity of grapiprant toward Caco-2 cells was evaluated using the CCK-8 assay. Cells were exposed to grapiprant at concentrations ranging from 1.56 to 50 μg/mL, prepared by two-fold serial dilution, for 6, 12, 24, and 48 h. No significant changes in cell viability were observed at any tested concentration or exposure time (Figure 2). These results indicate that grapiprant exerted no detectable cytotoxic effects within the tested concentration range, supporting the use of these concentrations in subsequent transport and cellular uptake studies.

### 3.4. Establishment of the Caco-2 Cell Model

#### 3.4.1. Morphological Characteristics of Caco-2 Cells

**Figure 3:**
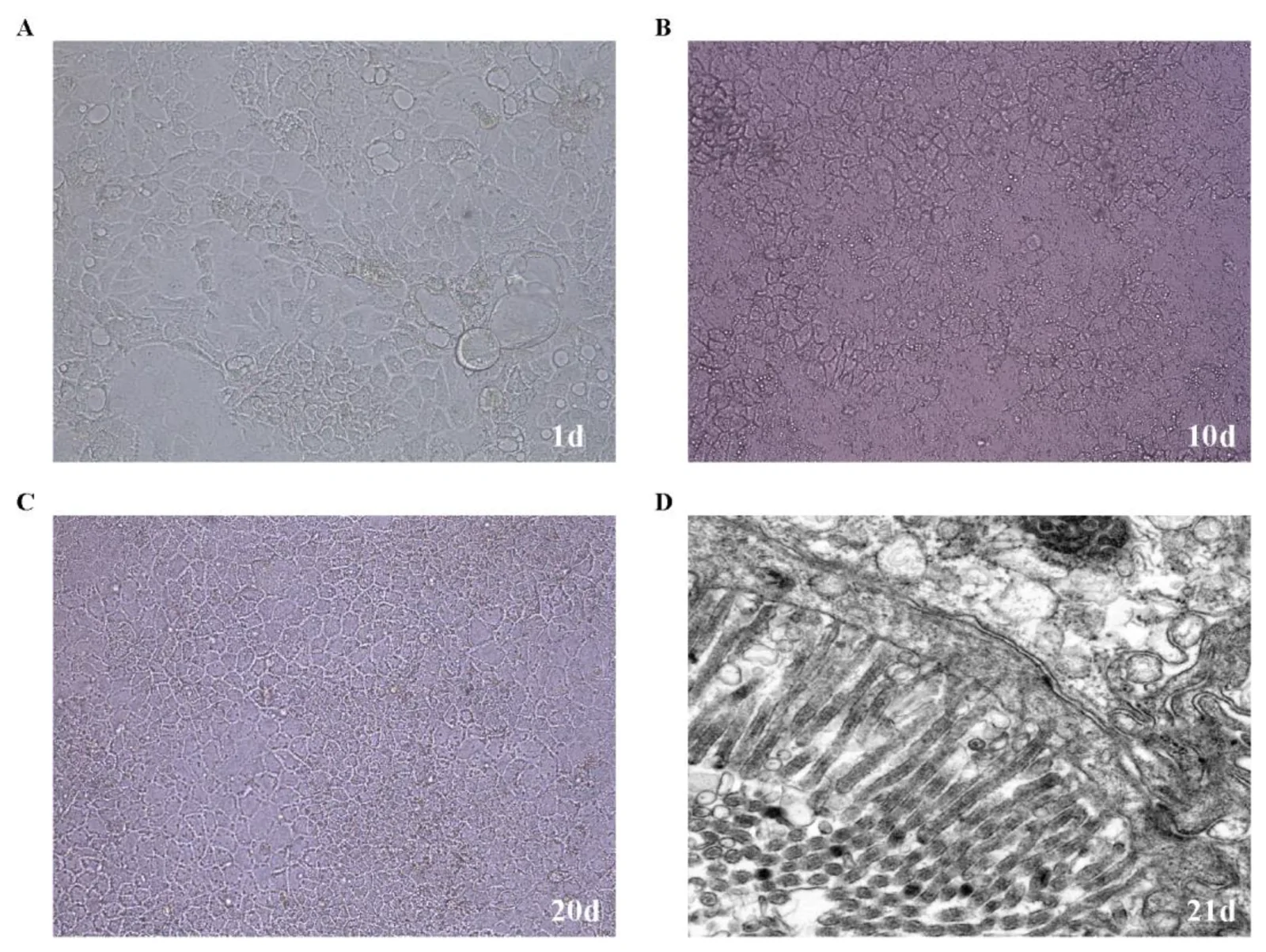
Morphology of Caco-2 cells during monolayer formation. (**A**–**C**) Light microscopy images after 1, 10, and 20 days of culture, respectively, using a 20× objective; (**D**) TEM image after 21 days of culture (scale bar: 500 nm).

Caco-2 cell morphology and growth were monitored daily by light microscopy. After 1 day of culture, the cells were sparsely distributed and had begun to attach (Figure 3A). After 10 days of culture, the cells had expanded markedly and approached confluence (Figure 3B). By day 20, they formed a compact, uniform, and continuous monolayer without visible intercellular gaps (Figure 3C). TEM showed regularly arranged microvilli on the apical surface of the cells after 21 days of culture (Figure 3D), consistent with the morphological characteristics of differentiated intestinal epithelial cells.

#### 3.4.2. TEER of Caco-2 Cell Monolayers

**Figure 4:**
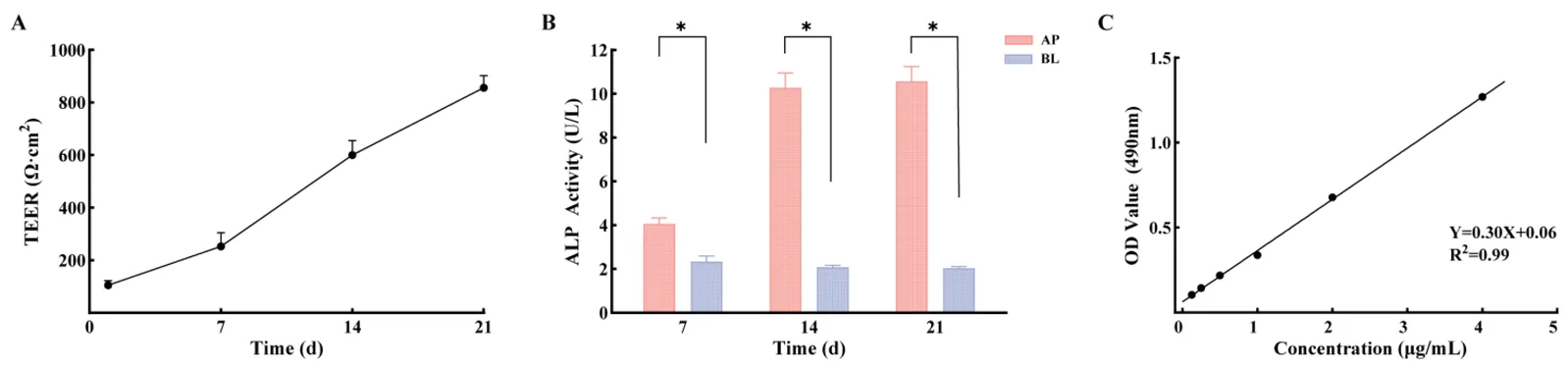
Validation of the Caco-2 cell monolayer model. (**A**) Changes in TEER over 21 days of culture; (**B**) ALP activity in the AP and BL sides on days 7, 14, and 21; (**C**) standard curve of sodium fluorescein based on absorbance at 490 nm. Significance test: At each time point, ALP activities on the AP and BL sides were compared using the Wilcoxon matched-pairs signed-rank test; * *p* < 0.05.

To evaluate the compactness and integrity of the Caco-2 cell monolayer, TEER values were measured every 7 days during culture. The TEER values increased progressively over the 21-day culture period and reached approximately 800 Ω·cm^2^ on day 21 (Figure 4A), indicating that the Caco-2 cell monolayer had developed satisfactory barrier integrity and tight intercellular junctions.

#### 3.4.3. Alkaline Phosphatase Activity

During the establishment of the Caco-2 model, the cells underwent epithelial differentiation and gradually formed a polarized monolayer with an apical brush border. ALP, a marker enzyme of the intestinal epithelial brush border, was used to evaluate the polarization and functional state of the monolayer. ALP activity in the AP and BL sides was measured at 7-day intervals. After 21 days of culture, ALP activity on the AP side was significantly higher than that on the BL side, with an AP/BL ratio of approximately 5 (Figure 4B). The asymmetric distribution of ALP indicated marked polarization of the Caco-2 monolayer and supported the successful establishment of the cell model.

#### 3.4.4. Permeability of Sodium Fluorescein

Sodium fluorescein was used as a marker to assess the permeability of the established Caco-2 cell monolayer model. The calibration curve was linear over the concentration range of 0.125–4 μg/mL, with the regression equation Y = 0.30X + 0.06 (R^2^ = 0.99) (Figure 4C). After 90 min, the sodium fluorescein concentration on the BL side was 0.40 ± 0.06 μg/mL, and the mean Papp _(AP-BL)_ was 4.91 × 10^−7^ cm/s (SD, 0.70 × 10^−7^; 95% CI, 4.46 × 10^−7^–5.36 × 10^−7^). This value was below the predefined acceptance criterion of 1 × 10^−6^ cm/s, indicating that the Caco-2 cell monolayer maintained sufficient integrity for subsequent absorption and transport experiments.

### 3.5. Bidirectional Transport Assay of Grapiprant in the Caco-2 Cell Monolayer Model

Cumulative transport was generally linear with time over the 15–90 min interval used for Papp calculation across the tested concentration, pH, and inhibitor conditions, with the corresponding R^2^ values provided in Supplementary Table S1.

#### 3.5.1. Effects of Time and Concentration on Grapiprant Transport in Caco-2 Cells

**Figure 5:**
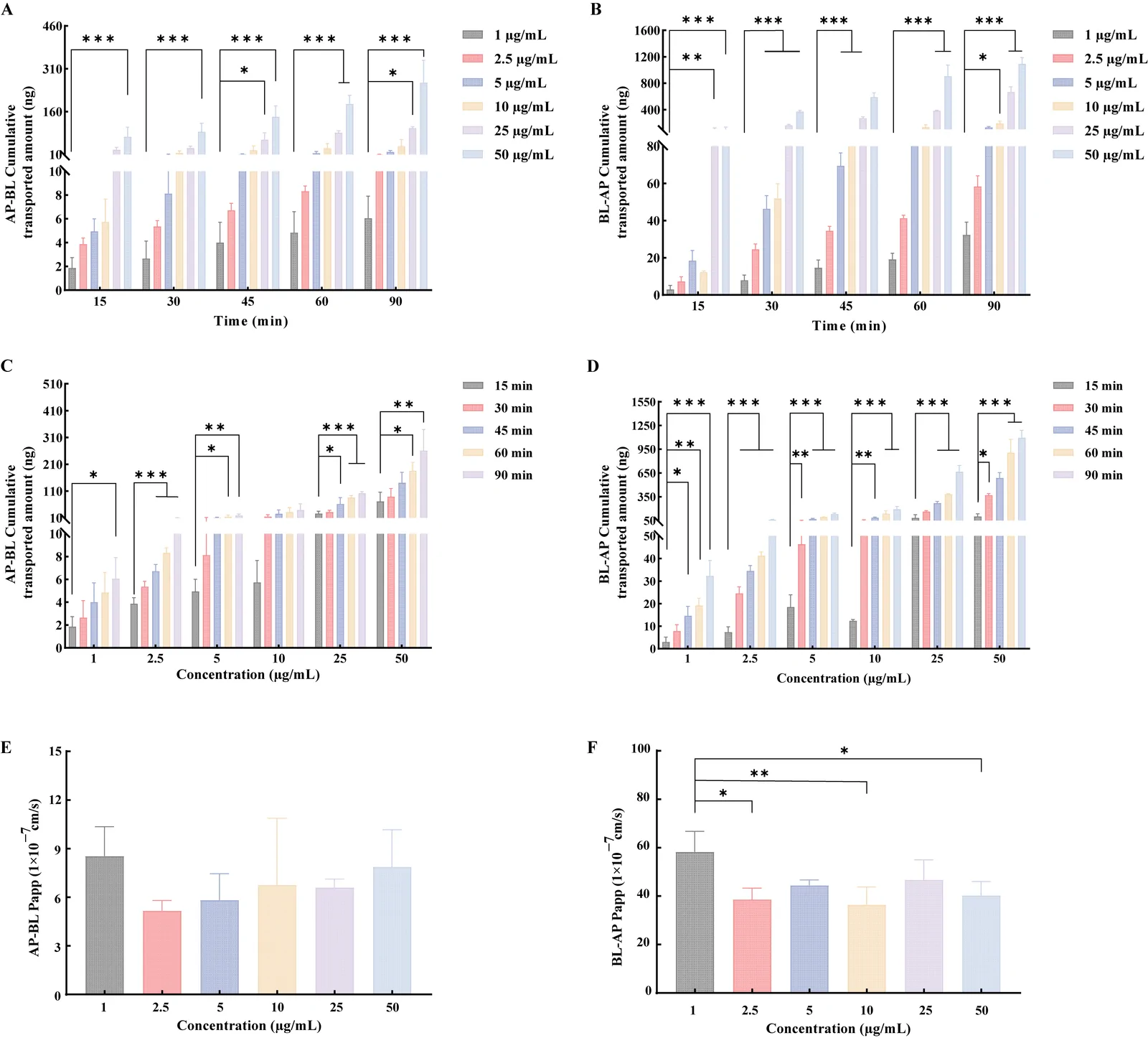
Effects of time and concentration on grapiprant transport across Caco-2 cell monolayers. (**A**,**C**,**E**) AP-BL transport; (**B**,**D**,**F**) BL-AP transport. (**A**,**B**) Cumulative amounts of grapiprant transported at different concentrations over 90 min; (**C**,**D**) cumulative amounts transported at different time points over the concentration range of 1–50 μg/mL; (**E**,**F**) Papp of grapiprant at different concentrations. Statistical analysis: One-way ANOVA followed by Dunnett’s multiple-comparisons test was used, with 1 μg/mL as the reference in (**A**,**B**,**E**,**F**) and 15 min as the reference in (**C**,**D**); * *p* < 0.05, ** *p* < 0.01, and *** *p* < 0.001.

The bidirectional transport characteristics of grapiprant across Caco-2 cell monolayers were evaluated at concentrations of 1, 2.5, 5, 10, 25, and 50 μg/mL over 90 min. The cumulative transport amount of grapiprant increased with both transport time and dosing concentration, indicating time- and concentration-dependent cumulative transport (Figure 5A,C). The mean Papp _(AP-BL)_ values ranged from 5.16 × 10^−7^ cm/s (SD, 0.65 × 10^−7^; 95% CI, 3.56 × 10^−7^–6.77 × 10^−7^) to 8.54 × 10^−7^ cm/s (SD, 1.82 × 10^−7^; 95% CI, 4.02 × 10^−7^–1.31 × 10^−6^). All mean Papp _(AP-BL)_ values were below 1 × 10^−6^ cm/s, indicating limited apparent permeability of grapiprant across the Caco-2 monolayer. The mean efflux ratio values of grapiprant at concentrations of 1–50 μg/mL ranged from 5.31 (SD, 1.04; 95% CI, 2.73–7.88) to 8.05 (SD, 2.23; 95% CI, 2.51–13.58). All mean efflux ratio values were markedly greater than 1, suggesting that the transmembrane transport of grapiprant may be influenced by efflux transporters.

#### 3.5.2. Effect of pH on Grapiprant Transport in Caco-2 Cells

**Figure 6:**
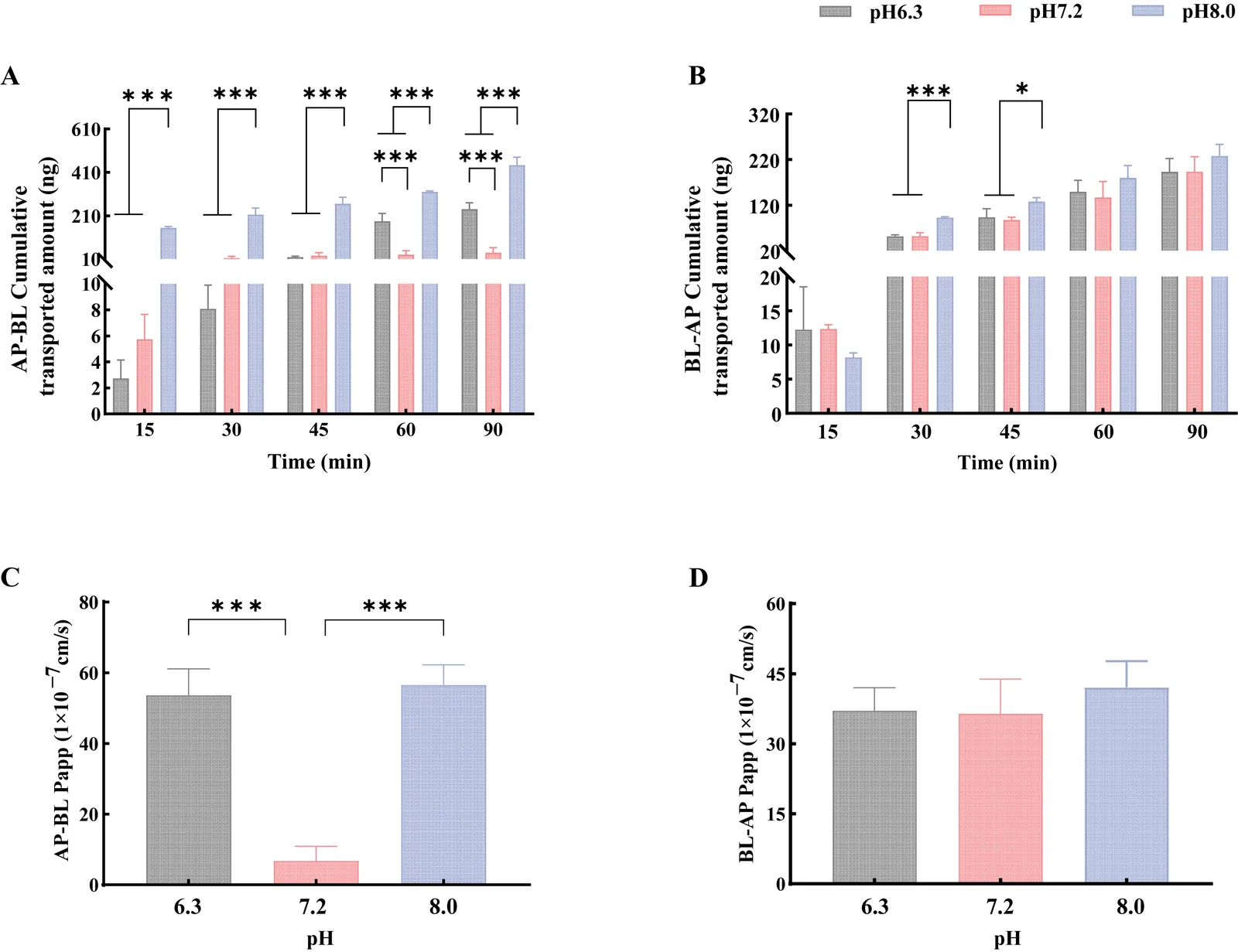
Effects of pH on grapiprant transport across Caco-2 cell monolayers. (**A**,**C**) AP-BL transport; (**B**,**D**) BL-AP transport. (**A**,**B**) Cumulative amounts of grapiprant transported at pH 6.3, 7.2, and 8.0 over 90 min; (**C**,**D**) Papp under different pH conditions. Significance test: One-way ANOVA followed by Tukey’s multiple-comparisons test was used, with pH groups in A and B compared at each time point; * *p* < 0.05 and *** *p* < 0.001.

The effect of pH on the transmembrane transport of grapiprant was further evaluated at pH 6.3, 7.2, and 8.0. In the AP-BL direction, the cumulative transport amount increased with incubation time from 15 to 90 min under all pH conditions and was significantly higher at pH 8.0 than at pH 6.3 or 7.2 (Figure 6A). The mean Papp _(AP-BL)_ values at pH 6.3 and 8.0 were 5.37 × 10^−6^ cm/s (SD, 0.75 × 10^−6^; 95% CI, 3.51 × 10^−6^–7.22 × 10^−6^) and 5.66 × 10^−6^ cm/s (SD, 0.57 × 10^−6^; 95% CI, 4.25 × 10^−6^–7.07 × 10^−6^), respectively, and both were significantly higher than the value at pH 7.2, which was 6.76 × 10^−7^ cm/s (SD, 4.12 × 10^−7^; 95% CI, −3.48 × 10^−7^–1.70 × 10^−6^) (Figure 6C). These results indicate that pH affects the transport of grapiprant across the Caco-2 cell monolayer, with mildly acidic and mildly alkaline conditions promoting its transport in the AP-BL direction.

#### 3.5.3. Effects of Efflux Inhibitors on Grapiprant Transport in Caco-2 Cells

**Figure 7:**
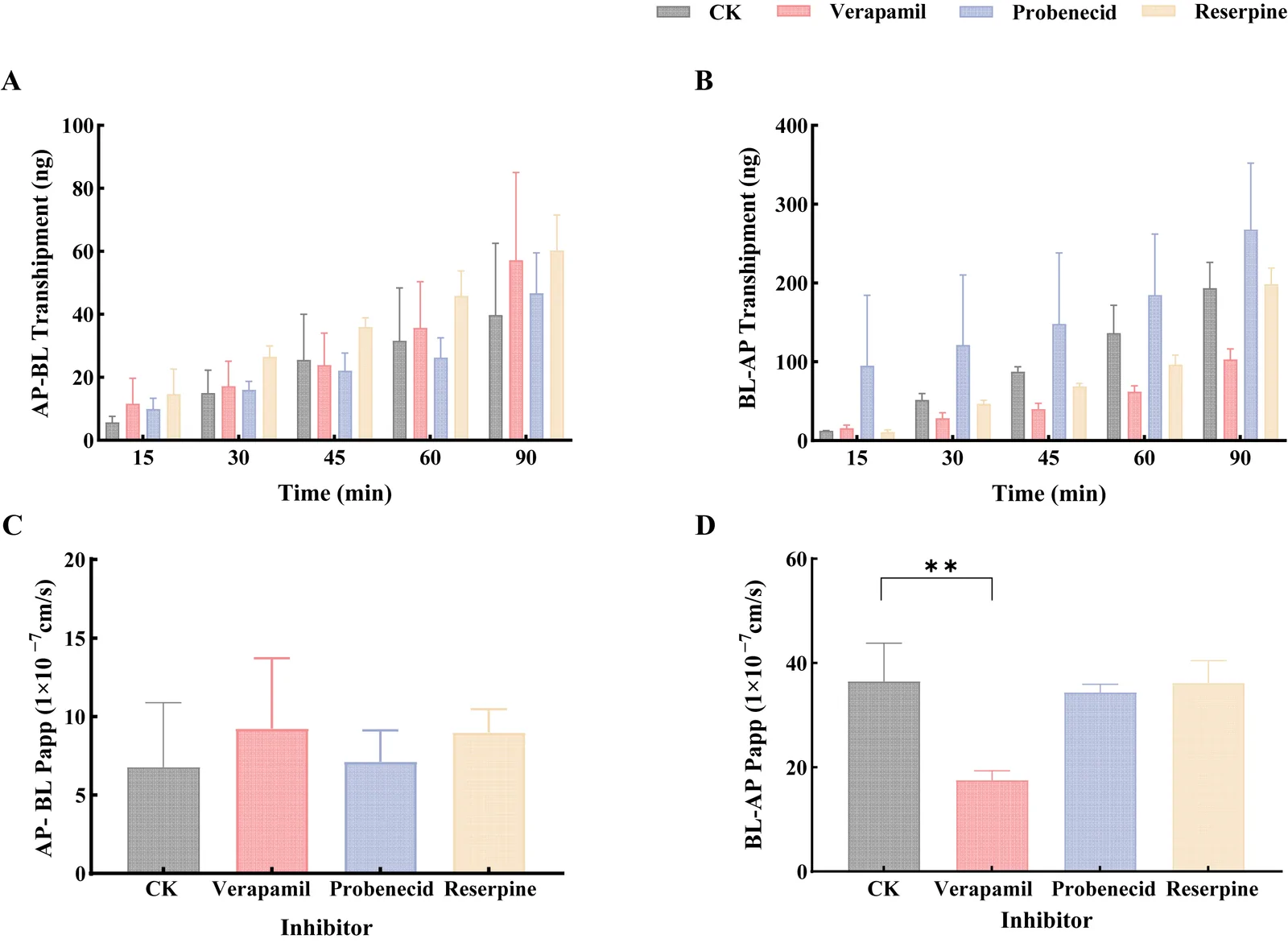
Effects of efflux transporter inhibitors on grapiprant transport across Caco-2 cell monolayers. (**A**,**C**) AP-BL transport; (**B**,**D**) BL-AP transport. (**A**,**B**) Cumulative amounts of grapiprant transported over 90 min; (**C**,**D**) Papp under different inhibitor treatments. Significance test: One-way ANOVA followed by Dunnett’s multiple-comparisons test was used, with the inhibitor-free control as the reference and inhibitor groups in A and B compared at each time point; ** *p* < 0.01.

To investigate the involvement of efflux transporters in the transmembrane transport of grapiprant, verapamil hydrochloride, probenecid, and reserpine were used as inhibitors of P-gp, MRP2, and BCRP, respectively. In the AP-BL direction, none of the three inhibitors significantly affected the cumulative transport amount or Papp of grapiprant (Figure 7A,C). In the BL-AP direction, verapamil significantly reduced the mean Papp from 3.65 × 10^−6^ cm/s (SD, 0.74 × 10^−6^; 95% CI, 1.82 × 10^−6^–5.48 × 10^−6^) to 1.75 × 10^−6^ cm/s (SD, 0.18 × 10^−6^; 95% CI, 1.31 × 10^−6^–2.20 × 10^−6^) and decreased the mean ER from 7.10 (SD, 4.78; 95% CI, −4.77–18.97) to 2.44 (SD, 1.65; 95% CI, −1.66–6.53), whereas probenecid and reserpine had no significant effects on Papp (Figure 7D). These results suggest that grapiprant may be a substrate of P-gp, whereas MRP2 and BCRP appear to have limited involvement in its efflux.

### 3.6. Uptake Assay and Mass Balance Recovery of Grapiprant in the Caco-2 Cell Monolayer Model

**Figure 8:**
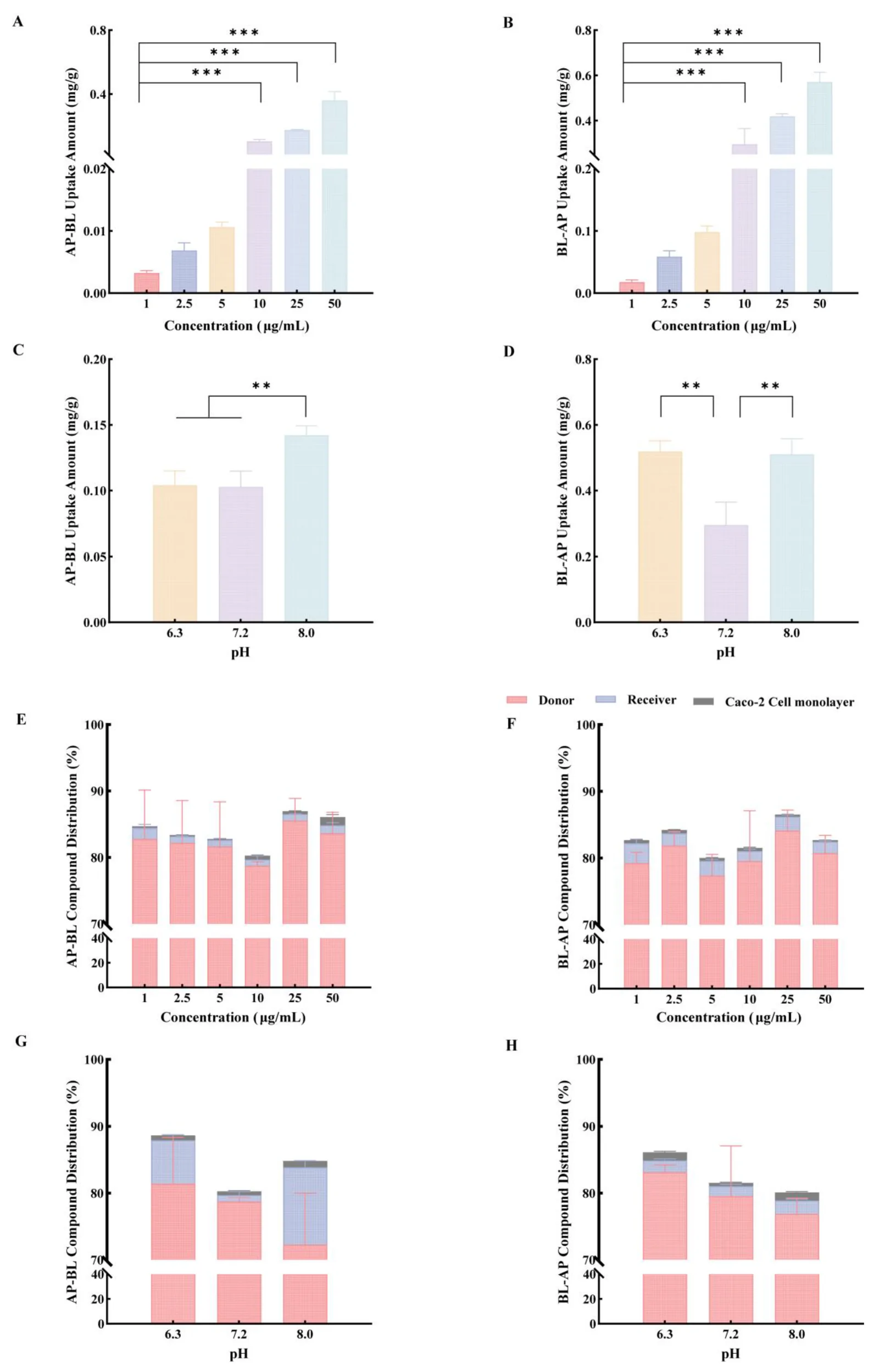
Cellular uptake and distribution of grapiprant following bidirectional transport across Caco-2 cell monolayers. (**A**,**C**,**E**,**G**) AP-BL transport; (**B**,**D**,**F**,**H**) BL-AP transport. (**A**,**B**) Cellular uptake at different concentrations; (**C**,**D**) cellular uptake under different pH conditions; (**E**,**F**) distribution among the donor side, receiver side, and Caco-2 cell monolayer at different concentrations; (**G**,**H**) distribution under different pH conditions. Statistical analysis: One-way ANOVA followed by Dunnett’s multiple-comparisons test was used for A and B, with 1 μg/mL as the reference, and Tukey’s multiple-comparisons test was used for C and D; ** *p* < 0.01 and *** *p* < 0.001.

After 90 min of transport, cellular uptake of grapiprant increased with donor concentration over the range of 1–50 μg/mL and was generally higher in the BL-AP direction than in the AP-BL direction (Figure 8A,B). Uptake also varied with pH, with higher levels observed at pH 8.0 in the AP-BL direction and lower levels at pH 7.2 in the BL-AP direction (Figure 8C,D).

Approximately 80% or more of the initial amount remained on the donor side, whereas less than 2% was detected on the receiver side and cellular uptake was generally below 1% (Figure 8E–H). These findings indicate limited transport of grapiprant across the Caco-2 monolayer, with cellular uptake contributing minimally to its overall distribution. Mass balance remained above 80% under all conditions, supporting acceptable recovery of grapiprant in the transport system.

## 4. Discussion

The BCS classifies drug substances according to their aqueous solubility and intestinal permeability [9]. Grapiprant, a selective EP4 receptor antagonist, is widely used in tablet form for the management of osteoarthritis-associated pain in dogs, but its biopharmaceutical properties within the BCS framework have not been fully characterized. Therefore, this study assessed the solubility of grapiprant under pH conditions relevant to the canine gastrointestinal tract and used a Caco-2 cell monolayer model to investigate its absorptive transport characteristics. The results demonstrated that the biopharmaceutical behavior of grapiprant varies under canine-relevant gastrointestinal conditions, suggesting that its BCS classification should be interpreted in a species-specific context.

The solubility assessment was designed based on BCS recommendations and the ionization characteristics of grapiprant. The tested conditions covered the physiologically relevant canine gastrointestinal pH range and included pH conditions corresponding to the pKa and pKa ± 1 [10,35,36,37,38]. Based on previous studies conducted in our laboratory, grapiprant showed two dissociation constants, with pKa_1_ and pKa_2_ values of 5.87 and 6.48, respectively, suggesting that it may exist in different ionization states within the canine gastrointestinal tract.

Grapiprant showed a modest variation in solubility across the tested pH range, with relatively lower solubility in the pKa_1_–pKa_2_ region compared with acidic and mildly alkaline conditions. This finding may be attributed to the ampholytic weak-electrolyte properties of grapiprant, which contains both acidic and basic ionizable groups. According to structure-based pKa prediction, the sulfonylurea NH is the main acidic ionizable site, with a predicted pKa of 5.55, whereas the pyridine-like nitrogen in the imidazopyridine ring is the main basic ionizable site, with a predicted pKa of 6.41. These predicted values were close to the experimentally determined pKa_1_ and pKa_2_ values of 5.87 and 6.48, respectively, suggesting that the two experimentally observed dissociation processes may be associated with these ionizable sites. Under acidic conditions, protonation of the basic nitrogen is favored, whereas at higher pH the sulfonylurea NH can become deprotonated. Around the pKa_1_–pKa_2_ region, both protonation and deprotonation processes may occur, resulting in a lower net molecular charge than under more acidic or alkaline conditions. A lower net charge around this region may contribute to the reduced aqueous solubility, which could explain the relatively lower solubility observed for grapiprant. Similar U-shaped pH–solubility profiles have been reported for zwitterionic fluoroquinolones, with minimum solubility occurring near the isoelectric point [39]. This pattern is consistent with the possibility that the relatively lower solubility of grapiprant around its pKa region is related to changes in its ionization state, although the specific distribution of ionic species was not experimentally determined in the present study.

Further D_0_ analysis showed that, at a fluid volume of 35 mL, D_0_ ranged from approximately 0.78 to 1.05 across the tested pH conditions. The lower solubility around the pKa_1_-pKa_2_ region corresponded to higher D_0_ values, approaching or slightly exceeding 1. Thus, although the variation in solubility was modest, it could still affect the D_0_-based solubility assessment when D_0_ was close to 1. At lower fluid volumes, the higher D_0_ values suggested greater potential for dissolution limitation. Clinically, limited gastric fluid or insufficient water intake may therefore reduce the amount of grapiprant dissolved, although the effect on systemic exposure requires further in vivo confirmation.

The fluid volumes and dose used for the D_0_ calculation were selected based on available canine physiological data and the clinical dosing regimen of grapiprant. Previous canine BCS studies used 6 and 35 mL as reference fluid volumes for approximately 10–11 kg Beagle dogs. The 6 mL value represented a lower estimate of residual gastric fluid in fasted dogs, whereas 35 mL was derived by scaling the 250 mL human BCS reference volume to a 10 kg Beagle dog [13,40]. In addition, an MRI study reported a mean fasted gastric fluid volume of 24.0 ± 4.2 mL in 9–12 kg Beagle dogs [36]; therefore, 24 mL was included as an intermediate reference volume based on direct in vivo measurement. The 20 mg dose is consistent with the labeled dosing regimen for this body-weight range, as dogs weighing 6.9–13.6 kg are recommended to receive one 20 mg grapiprant tablet once daily. Although higher tablet strengths are used in larger dogs, extending the analysis to other body-weight and dose scenarios would require reliable estimates of gastric fluid volume for dogs of different sizes. Available data are insufficient to define how gastric fluid volume changes with canine body weight, and simple linear scaling by body weight may produce unrealistic estimates, particularly in large dogs [41]. Therefore, based on the available physiological and clinical data, the present D_0_ analysis was performed using a 20 mg dose and the literature-supported gastric fluid volumes described above.

To characterize the intestinal permeability of grapiprant, a Caco-2 monolayer model was used to mimic the intestinal epithelial barrier. The monolayer was validated before transport experiments by TEER measurement, TEM observation, ALP activity, and sodium fluorescein transport, confirming its integrity, polarity, and barrier function and supporting the reliability of the permeability data [28,42,43].

However, species-related differences exist between human-derived Caco-2 cells and the canine intestinal epithelium. The TEER values of canine colonoid-derived monolayers were reported to be approximately eightfold higher than those of Caco-2 monolayers, indicating tighter intercellular junctions. Canine colonoid-derived monolayers also contained mucus-producing goblet cells and showed differences in intestinal transporter expression, including higher MDR1 expression [44,45]. Although canine intestinal organoid-derived monolayers are more relevant to canine intestinal physiology, their application to drug permeability assessment is still at an early stage. Establishing these models requires primary canine intestinal tissue, specialized extracellular matrices and growth-factor-supplemented media, followed by the expansion and differentiation of three-dimensional organoids and further optimization to obtain reproducible monolayers on permeable supports. In addition, characteristics of canine intestinal organoids may vary according to the intestinal segment of origin, while the procedures used for their culture and permeability assessment remain less standardized than those used for the Caco-2 model [45,46,47,48].

Therefore, given the well-established use, controllable experimental conditions, and good reproducibility of the Caco-2 model, it was used in this study to compare grapiprant transport under different experimental conditions and to examine its potential efflux mechanisms. However, the measured Papp values reflect the apparent permeability of grapiprant only within the Caco-2 model. Because canine-specific Papp thresholds have not been established, these values were described qualitatively as indicating limited or moderate permeability rather than being used to estimate the absolute fraction absorbed in dogs.

The permeability results showed that the Papp _(AP-BL)_ values of grapiprant did not differ significantly across the concentration range, suggesting no apparent concentration-dependent saturation. However, the overall Papp _(AP–BL)_ values were below 1 × 10^−6^ cm/s, and most of the drug remained on the donor side, indicating limited apparent permeability of grapiprant across the Caco-2 monolayer [43]. In addition, the efflux ratio (ER > 1) suggested that the transepithelial transport of grapiprant may be influenced by active efflux mechanisms.

The effect of intestinal pH on grapiprant transport was further evaluated at pH 6.3, 7.2, and 8.0, corresponding to the canine duodenum, jejunum, and ileum, respectively. The results showed that intestinal pH altered the permeability behavior of grapiprant. For ampholytic compounds, ionized forms generally favor aqueous solubility, whereas unionized or less charged species may show greater membrane partitioning and diffusional potential. Therefore, the pH-dependent differences in grapiprant transport may reflect a dynamic balance between solubility and membrane partitioning, rather than being determined solely by changes in solubility.

Bidirectional transport experiments showed a relatively high ER for grapiprant. Verapamil significantly reduced BL-AP transport and the ER, whereas the MRP2 and BCRP inhibitors had no significant effects. Because Caco-2 cells are human-derived, these findings suggest that P-gp may contribute to the efflux of grapiprant in this model and are consistent with the previous identification of grapiprant as a human P-gp substrate. P-gp is encoded by the ABCB1/MDR1 gene and is expressed in barrier and excretory tissues in dogs, including the intestinal epithelium, hepatobiliary system, kidney, and blood–brain barrier [49]. Therefore, alterations in P-gp function may affect the absorption, distribution, and clearance of grapiprant.

The potential in vivo relevance of P-gp is further supported by a pharmacokinetic study in MDR1-1Δ homozygous Collies, in which systemic exposure to grapiprant was substantially higher than that reported in dogs with normal P-gp function [32]. However, the increased systemic exposure in these dogs cannot be attributed solely to enhanced intestinal absorption. Although P-gp deficiency may theoretically increase oral bioavailability, the increased exposure to grapiprant in these dogs may reflect the combined effects of intestinal, hepatic, and biliary disposition processes rather than increased absorption alone.

In the present Caco-2 experiments, the ER remained greater than 1 after verapamil treatment. A similar pattern has been reported in Caco-2 cells, where the ER of digoxin decreased from 9.53 to 1.49 after treatment with the ABCB1 inhibitor CP100356 [50]. This residual ER does not exclude the involvement of P-gp in the efflux process. In addition, Papp and ER measured in bidirectional monolayer assays can be influenced by transport resistances associated with the experimental system, including aqueous boundary layers and the filter supporting the cell monolayer, as well as by paracellular transport [51]. Therefore, the ER remaining above 1 after verapamil treatment in the present study may reflect incomplete P-gp inhibition or the contribution of other transport processes.

A limitation of the present study is that only one inhibitor was used for each transporter. Further evaluation of P-gp involvement could combine several complementary approaches, including the use of more selective inhibitors [52], measurement of ABCB1 mRNA and P-gp protein expression in the Caco-2 cells [53], and functional validation with established P-gp probe substrates [50,53]. Previous studies have shown that these approaches can provide additional information on transporter expression and activity, while the use of different probe substrates may also help reduce uncertainty associated with a single functional assay. Therefore, the present results support the possible involvement of P-gp in grapiprant efflux in the Caco-2 model, but are not sufficient to establish grapiprant as a P-gp substrate.

Overall, under canine-relevant gastrointestinal conditions, both solubility and intestinal permeability may influence the oral absorption of grapiprant. Accordingly, future optimization should consider both dosing conditions and formulation design. Adequate fluid intake during administration may increase the amount of grapiprant dissolved in the gastrointestinal tract, although whether this translates into greater systemic exposure requires further pharmacokinetic confirmation. This could be evaluated by comparing systemic exposure after administration of the same grapiprant tablet with different volumes of water under standardized fasting conditions [54,55,56]. Alternatively, the current tablet could be compared with formulations designed to improve dissolution to determine whether changes in dissolution are accompanied by changes in systemic exposure [30,57,58]. Among potential formulation approaches, nanocrystal formulations and amorphous solid dispersions (ASDs) may be considered because both have been widely used to improve the dissolution of poorly water-soluble drugs and can be developed as solid oral dosage forms [59,60,61]. Their suitability for grapiprant, however, requires further experimental evaluation.

## 5. Conclusions

These findings indicate that the application of the BCS framework to veterinary drugs should account for species-specific gastrointestinal conditions. For grapiprant, both solubility and intestinal permeability may contribute to limiting oral absorption under certain canine-relevant conditions. grapiprant, limited apparent permeability may become more relevant when solubility is favored by acidic pH or larger gastric fluid volumes, whereas lower gastric fluid volumes may increase the contribution of solubility limitation to oral absorption. Accordingly, the present study provides a biopharmaceutical characterization of grapiprant within the BCS framework rather than a definitive canine BCS classification. Optimization of dosing conditions and formulation strategies may improve its oral absorption, although further in vivo pharmacokinetic studies in dogs are required to verify these potential effects.

## Figures and Tables

**Table 1:** Solubility of grapiprant under different pH conditions.

pH	Solubility (μg/mL)
1.6	732.07 ± 5.53
3.8	697.54 ± 8.00
4.87	650.23 ± 14.59
5.48	587.96 ± 11.72
5.87	546.84 ± 19.22
6.48	550.98 ± 11.19
6.87	564.33 ± 5.87
7.48	584.21 ± 5.24
8.0	686.94 ± 1.21

**Table 2:** D_0_ of grapiprant under different pH conditions.

Gastric Volume (mL)	Dose (mg)	pH								
		1.6	3.8	4.87	5.48	5.87	6.48	6.87	7.48	8.0
6	20	4.55	4.78	5.13	5.67	6.10	6.05	5.91	5.71	4.85
24		1.14	1.19	1.28	1.42	1.53	1.51	1.48	1.43	1.21
35		0.78	0.82	0.88	0.97	1.05	1.04	1.01	0.98	0.83

## Data Availability

The data presented in this study are available on request from the corresponding author.

## Supplementary Materials

The following supporting information can be downloaded at: https://www.mdpi.com/article/10.3390/pharmaceutics18081010/s1, Table S1. Linearity of cumulative grapiprant transport versus time under different experimental conditions.
