# Supplementary material for: Biopharmaceutical Characterization of Grapiprant Within the BCS Framework Under Canine-Relevant Conditions: Solubility and Caco-2 Permeability Assessment
Source: Pharmaceutics. 2026 Aug 15;18(8):1010. doi: 10.3390/pharmaceutics18081010 (PMC13516831; doi:10.3390/pharmaceutics18081010)
Supplement: Supplementary file 1 [file pharmaceutics-18-01010-s001.zip › pharmaceutics-4484948-supplementary.pdf]

Table S1. Linearity of cumulative grapiprant transport versus time under different experimental conditions.

Experimental factor	Condition	Direction	R^2 (mean ± SD)
Concentration	1 µg/mL	AP-BL	0.909±0.042
		BL-AP	0.993±0.004
	2.5 µg/mL	AP-BL	0.977±0.014
		BL-AP	0.950±0.061
	5 µg/mL	AP-BL	0.961±0.019
		BL-AP	0.983±0.018
	10 µg/mL	AP-BL	0.921±0.055
		BL-AP	0.987±0.013
	25 µg/mL	AP-BL	0.863±0.059
		BL-AP	0.984±0.011
	50 µg/mL	AP-BL	0.931±0.078
		BL-AP	0.935±0.048
pH	pH6.3	AP-BL	0.854±0.034
		BL-AP	0.962±0.042
	pH7.2	AP-BL	0.921±0.055
		BL-AP	0.987±0.013
	pH8.0	AP-BL	0.980±0.007
		BL-AP	0.923±0.040
Inhibitor	Verapamil	AP-BL	0.967±0.016
		BL-AP	0.981±0.012
	Probenecid	AP-BL	0.969±0.007
		BL-AP	0.976±0.015
	Reserpine	AP-BL	0.972±0.027
		BL-AP	0.969±0.012

Table S1. Values are presented as mean ± SD (n = 3). R^2 was obtained by linear regression of cumulative transported amount versus time over the 15-90 min interval used to determine dQ/dt. AP-BL, apical to basolateral side; BL-AP, basolateral to apical side.
